# Increased Number of Human Cases of Influenza Virus A(H5N1) Infection, Egypt, 2014–15

**DOI:** 10.3201/eid2112.150885

**Published:** 2015-12

**Authors:** Samir Refaey, Eduardo Azziz-Baumgartner, Marwa Mohamed Amin, Manal Fahim, Katherine Roguski, Hanaa Abu Elsood Abd Elaziz, A. Danielle Iuliano, Noha Salah, Timothy M. Uyeki, Steven Lindstrom, Charles Todd Davis, Alaa Eid, Mohamed Genedy, Amr Kandeel

**Affiliations:** Egyptian Ministry of Health, Cairo, Egypt (S. Refaey, M.M. Amin, M. Fahim, H.A.E.A. Elaziz, N. Salah, A. Eid, M. Genedy, A. Kandeel);; Centers for Disease Control and Prevention, Atlanta, Georgia, USA (E. Azziz-Baumgartner, K. Roguski, A.D. Iuliano, T.M. Uyeki, S. Lindstrom, C.T. Davis)

**Keywords:** influenza, viruses, avian influenza, H5N1, human, poultry, Egypt

## Abstract

During November 2014–April 2015, a total of 165 case-patients with influenza virus A(H5N1) infection, including 6 clusters and 51 deaths, were identified in Egypt. Among infected persons, 99% reported poultry exposure: 19% to ill poultry and 35% to dead poultry. Only 1 person reported wearing personal protective equipment while working with poultry.

Highly pathogenic avian influenza virus A(H5N1) has been detected among poultry in >60 countries, with sporadic transmission to humans that results in a large number of deaths (*1*). Of 842 persons with H5N1 virus infection reported as of June 23, 2015, worldwide, 447 (53%) died (*2*,*3*). During November 2014–February 2015, the Egyptian Ministry of Health (MoH) surveillance systems identified an unprecedented number of persons with severe respiratory illness caused by infection with H5N1 virus. These illnesses occurred during months when seasonal influenza is typically epidemic in Egypt (*4*). In response, the MoH initiated an investigation into potential causes of the increased number of cases.

## The Study

Since 2006, MoH mandates that clinicians refer all persons with influenza-like illness (ILI) and <2-week history of poultry contact to 1 of ≈83 Chest and Fever hospitals (i.e., a category of referral hospitals) throughout Egypt (Technical Appendix Figure). Persons meeting the ILI case definition have fever >38°C and >1 of the following: cough, dyspnea, sore throat, myalgia, and body aches. Persons meeting the ILI case definition are admitted, and respiratory samples are collected for influenza testing.

Technicians at 8 sentinel sites also collected daily respiratory samples from 2 patients meeting the ILI case definition and from all patients admitted with severe acute respiratory infection, defined as hospitalization occurring within 2 weeks of onset of fever and cough. Nasal and oropharyngeal swabs were transported to Egypt’s National Influenza Center for testing by reverse transcription PCR (*2*). For patients testing positive for H5N1, MoH staff visited their households; administered a standardized questionnaire to obtain demographic, exposure, clinical, and treatment information; and searched among patient contacts for additional case-patients.

We obtained surveillance data collected during 2006–2015 and compared case-patients with H5N1 virus infection for November 2014–April 2015 to those reported in previous years. We obtained the average epidemic period by calculating the proportion of samples testing positive for H5N1 each month throughout the period (*4*). An epidemic was defined as consecutive months having a proportion of H5N1-positive samples that exceeded the annual July–June median. 

During March 20, 2006–April 20, 2015, a total of 342 persons with H5N1 virus infection were identified in Egypt (Figure). Annual epidemics typically occurred during November–April, when 299 (87%; 95% CI 84%–91%) of the 342 H5N1 illnesses occurred, resulting in a median of 23 case-patients (interquartile range [IQR] 13–31) per epidemic. Of the total 342 case-patients, 165 were identified during 2014–15, including 6 clusters of 2–3 case-patients. Although this season had a higher number of case-patients than previous years, other seasons had higher proportions of H5N1 detections or clusters among samples tested. The median percentage (5.4%) of monthly H5N1 detections among humans sampled during November 2014–April 2015 was statistically similar to the median percentage (2.9%) for March 2006–October 2014 (Kruskal-Wallis test; p = 0.5). The proportion of clustered case-patients was also similar for the 2 periods: 12 (7.3%; 95% CI 3.3%–11.2%) of 165 case-patients during 2014–15 and 12 (6.8%; 95% CI 3.1–10.5) of 177 case-patients during 2006–2014. The number of human H5N1 case-patients identified each month was highly correlated with the number of H5N1 poultry outbreaks identified each month during 2006–2015 by the Ministry of Agriculture in the same communities (R = 0.3, p = 0.002).

**Figure:**
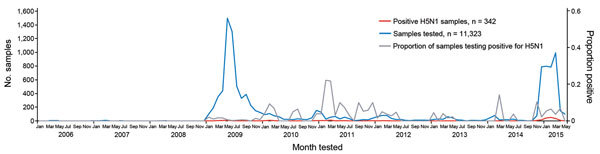
Number of human respiratory samples tested, number of samples testing positive for influenza virus A(H5N1), and proportion of positive samples, Egypt, March 2006–May 2015.

The case-patients identified during 2014–15 were characteristically similar to those from previous seasons. Similar percentages were found to be women (100/165 [61%] for 2014–15; 105/177 [59%] for 2006–2014), homemakers (50/164 [30%] for 2014–15; 46/177 [26%] for 2006–2014), and poultry farm workers (2/165 [1%] for 2014–15; 0 for 2006–2014). Case fatality rates were also similar: 51 (31%) deaths among 165 case-patients during 2014–15 versus 64 deaths (36%) among 177 case-patients for March 2006–October 2014 (p = 0.3).

Almost all (163/165) case-patients during 2014–15 had exposure to domestic poultry 1–2 weeks before symptom onset; 58% were involved in breeding, 24% in slaughtering, and 21% in preparing poultry. Most (115/165 [70%]) were exposed at home; 4% recalled exposure in shops, 3% at live bird markets, and 1% at farms. Although 35% recalled exposure to dead birds and 19% to ill birds, 35% recalled exposure only to birds that appeared healthy. One case-patient reported wearing personal protective equipment when working with poultry.

Case-patients identified in 2014–15 were admitted and received oseltamivir treatment a median of 4 (IQR 2–7) days after symptom onset. Sixteen percent had preexisting medical conditions: 8 (5%; 95% CI 2%–8%) had chronic chest illnesses; 5 (3%; 95% CI 0.3%–6%) had cardiovascular disease; 5 (3%; 95% CI 0.3%–6%) had diabetes; 2 (1%; 95% CI 0%–3%) had renal failure; 2 (0.1%; 95% CI 0%–3%) had liver failure; and 1 (0.6%; 95% CI 0%–2%) was obese. In addition, 9 (10%; 95% CI 4%–16%) of 92 women 15–49 years of age were pregnant. Most case-patients had cough (87%) and dyspnea (72%) during their illness.

As of July 2015, of the 165 case-patients, 114 (69%) had survived and 51 (31%) died. Survivors were younger than decedents (median age 16 [IQR 3–36] vs. 33 [IQR 20–43] years; p = 0.0001). Oseltamivir treatment was begun within a median 4 (IQR 2–6) days of symptom onset for survivors, compared with a median 5 (IQR 3–7) days for decedents (2-sided Wilcoxon rank-sum test; p = 0.07).

## Conclusions

Our analyses suggest that H5N1 infections have recurred annually in Egypt during November–April. Although MoH identified an unprecedented number of H5N1 case-patients during 2014–15, the proportion of persons testing positive was similar to proportions of previous epidemic seasons. During 2006–2015, the Ministry of Agriculture identified 3,273 outbreaks among poultry, primarily during Egypt’s November–April winter months (*1*). One study found that ≈2% of Egyptians exposed to poultry were seropositive for H5N1 virus (*5*). The large number of H5N1 case-patients identified during 2014–15 could result in part from increased respiratory sampling in communities with poultry outbreaks, rather than from marked changes in the virus’s transmission characteristics.

The H5N1 case-patients during the 2014–15 season had similar characteristics to those of previous seasons (*6*). Nearly all had recent exposure to domestic poultry (*7*). Active surveillance from 2010–2012 suggests that 8% of healthy-appearing poultry in Egypt were infected with H5N1 clade 2.2.1 (*8*), yet only 1 case-patient in 2014–15 reported using personal protective equipment.

Human H5N1 infections have been shown to occur during poultry outbreaks, overlapping with October–December influenza epidemics (*4*). Egypt currently recommends seasonal influenza vaccination among health care workers, pregnant women, persons with chronic diseases, and Hajj and Umrah travelers. Countries where seasonal influenza overlaps with H5N1 circulation in poultry might explore the feasibility of vaccinating persons at high risk for influenza co-infections and complications (*9*).

After identification of case-patients in Egypt, officials investigated contacts. This strategy perhaps enriched the number of H5N1 case-patients identified during peak epidemic months, compared with randomly selecting persons meeting case definitions for respiratory illnesses. Although we did not find increased rates of persons testing positive for H5N1, all H5N1 case-patients are unlikely to have the same probability of being identified (i.e., contacts vs. randomly selected persons). 

Egypt continues to have substantial H5N1 circulation among poultry. Although the characteristics of case-patients during 2014–15 were similar to those of previous seasons and do not suggest increased efficiency of H5N1 transmission between humans, MOH would be warranted in examining H5N1 virus circulating in Egypt for genomic markers of mammalian adaptation (*10*), which have been identified since 2010 (*11*), and in using a cross-sectoral approach to evaluate interventions to prevent H5N1 infections.

**Technical Appendix.** Figure showing locations of human cases of influenza virus A(H5N1) infection, clusters of cases, and Chest and Fever hospitals where case-patients were treated, Egypt, November 2014–April 2015.
