# Supplementary material for: Increased Number of Human Cases of Influenza Virus A(H5N1) Infection, Egypt, 2014–15
Source: Emerg Infect Dis. 2015 Dec;21(12):2171–3. doi: 10.3201/eid2112.150885 (PMC4672432; doi:10.3201/eid2112.150885)
Supplement: Supplementary file 1 — Technical Appendix. Figure showing locations of human cases of influenza virus A(H5N1) infection, clusters of cases, and Chest and Fever hospitals where case-patients were treated, Egypt, November 2014–April 2015. [file 15-0885-Techapp-s1.pdf]

Article DOI: http://dx.doi.org/10.3201/eid2112.150885

Increases in Number of Human Cases of Influenza Virus A(H5N1) Infection, Egypt, 2014–2015

Technical Appendix

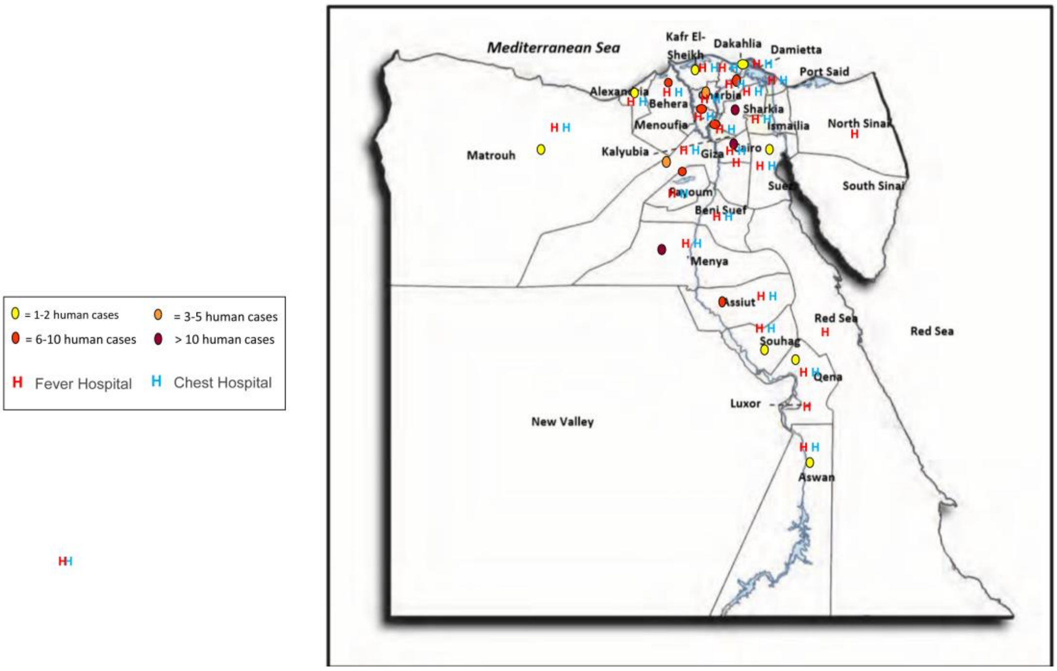

Technical Appendix Figure. Location of human cases, clusters of cases, and Chest and Fever hospitals that treated case-patients with influenza virus A(H5N1) infections, Egypt, November 2014–April 2015. Total case-patients = 165.
